# Vitamin K status has no influence on the effect of vitamin D supplementation on bone turnover and cardiovascular markers: a randomized controlled trial

**DOI:** 10.3389/fnut.2026.1857443

**Published:** 2026-06-30

**Authors:** Verena Theiler-Schwetz, Uwe Riedmann, Sieglinde Zelzer, Andreas Meinitzer, Lisa Schmitt, Daniel Arian Kraus, Christian Trummer, Martin R. Grübler, Martin H. Keppel, Armin Zittermann, Andreas Tomaschitz, Winfried März, Pawel Pludowski, Stefan Pilz

**Affiliations:** 1 Division of Endocrinology and Diabetology, Department of Internal Medicine, Medical University of Graz, Graz, Austria; 2 Clinical Institute of Medical and Chemical Laboratory Diagnostics, Medical University of Graz, Graz, Austria; 3 University Hospital Wiener Neustadt, Wiener Neustadt, Austria; 4 ENML—Erste NÖ Medizinische Laborbetriebs GmbH, Pölten, Austria; 5 Clinic for Thoracic and Cardiovascular Surgery, Herz und Diabeteszentrum Nordrhein-Westfalen (NRW), Ruhr University Bochum, Bad Oeynhausen, Germany; 6 Facharzt-Ordination Innere Medizin, Bruck an der Mur, Austria; 7 SYNLAB Academy, Synlab Holding Deutschland GmbH, Mannheim, Germany; 8 Medical Clinic III (Cardiology, Pneumology, Angiology) University of Heidelberg, Mannheim, Germany; 9 Medical Clinic (Cardiology), Medical University of Graz, Austria; 10 Department of Clinical Biochemistry, The Children's Memorial Health Institute, Warsaw, Poland

**Keywords:** bone turnover markers, cardiovascular, menaquinones, vitamin D, vitamin K

## Abstract

**Background/objectives:**

Vitamin K and D co-treatment may have additive effects on bone and cardiovascular health and is frequently used as an over-the-counter supplement. The primary aim of this study was to assess whether the effect of vitamin D supplementation on bone turnover markers and cardiovascular parameters is modified according to vitamin K status at baseline (i.e., serum levels of vitamin K1, MK4 and MK7).

**Methods:**

This is a post-hoc analysis of a randomized controlled vitamin D supplementation (2,800 international units daily for 8 weeks) trial in 200 participants. We measured vitamin K1, MK4 and MK7 by means of the currently available gold standard liquid chromatography tandem mass spectrometric and analysed cardiovascular and bone markers (including 24-h systolic and diastolic blood pressure, glucose and lipid parameters as well as osteocalcin, beta-crosslaps, and procollagen type 1 N-terminal propeptide). The main aim was to analyze whether pre-intervention vitamin K status (moderator) influenced the effects of vitamin D supplementation on outcome measures of bone and cardiovascular health after the intervention (moderation analyses). We also investigated whether vitamin D supplementation effects vitamin K status and whether vitamin K measures correlated with the outcome parameters.

**Results:**

Moderation analyses showed no significant interaction between vitamin K status and the intervention (vitamin D supplementation/placebo) on cardiovascular or bone parameters. We observed no treatment effect of vitamin D on vitamin K status. In exploratory analyses, parameters of vitamin K status correlated only weakly and inconsistently with some bone and cardiovascular parameters.

**Conclusion:**

The data from this post-hoc analysis do not provide evidence for the assumption that vitamin K status modifies or enhances effects of vitamin D supplementation on cardiovascular or bone parameters. These findings are in line with current guidelines, which do not routinely recommend vitamin K and D co-treatment in patients with osteoporosis. However, further trials are needed to evaluate the potential benefits of vitamin K supplementation and its combination with vitamin D.

## Introduction

In addition to calcium and vitamin D, vitamin K2 is increasingly used as an over-the-counter (OTC) supplement and prescribed to patients with osteoporosis owing to suggestive data related to bone mineral density (BMD) and fracture risk. Nevertheless, current guidelines do not recommend its routine use due to the heterogeneity of the existing literature and lack of conclusive evidence from high-quality clinical trials (1–4).

Vitamin K belongs to a family of fat-soluble compounds including phylloquinone (vitamin K1) and a series of menaquinones (vitamin K2). Vitamin K1 is the predominant dietary form found in green vegetables and is important for the synthesis and carboxylation of several vitamin K-dependent coagulation factors. Vitamin K2, most commonly present in the form of menaquinones 4 to 10 (MK-4 to MK-10, respectively), is an essential cofactor for the carboxylation of glutamic acid (Glu) in vitamin K dependent proteins (VKDPs), including osteocalcin (OC) and matrix glutamate (Gla) protein (MGP) (5–7). Especially fermented soy products like natto are rich in vitamin K2 (8). MK-7 compared to MK-4 has an extended half -life time (approximately 3 days versus 1 h) and might be effective in much lower amounts, which is the reason for using MK-7 in supplements (9).

OC promotes the import of calcium from the blood and several tissues into the bone for incorporation into hydroxyapatite, enhancing bone mineralization (10, 11) Vitamin K2 (MK-4) supplementation and vitamin K1 intake might improve lumbar spine BMD and increase bone strength (12), evidence from studies on bone turnover markers, BMD and fracture risk is conflicting, though (13–23).

Similarly, heterogenous data have pointed towards an association of vitamin K with cardiovascular health. Vitamin K, in particular vitamin K2, is known for its calcium homeostasis function and its deficiency seems to be responsible for the so-called “calcium paradox” phenomenon, defined by low calcium deposition in the bone and accumulation of mineral matrix in the vessel wall, known as vascular calcification (VC) (24). Vitamin K might mitigate vascular calcification in some (25–27), but not all studies (28), and could be associated with reduced mortality (29) by enhancing the activity of MGP (30, 31). The inactive forms of MGP, particularly dephosphorylated-uncarboxylated MGP (dp-ucMGP), could be a marker for vascular vitamin K status, and calcification and cardiovascular risk.

Poor skeletal health, cardiovascular events and mortality have also been related to a deficiency in vitamin D (32). Vitamin D has multiple roles beyond calcium homeostasis due to the ubiquitous expression of the vitamin D receptor (33). Due to its potential to lower the risk of mortality, current Endocrine Society Guidelines on vitamin D suggest empiric vitamin D supplementation in the general population aged 75 years and older (34). While the Endocrine Society Clinical Practice Guideline itself does not mention the upper dose limit of empiric vitamin D supplementation, it does endorse the Institute of Medicine report which recommends 4,000 IU per day as the upper intake level of vitamin D for individuals aged 9 years and older (35).

Preclinical studies have shown that the combination of vitamin D and K might exert a synergistic action by generating higher levels of bone anabolic markers and calcium deposits in osteoblasts (36). Further, vitamin K and D co-treatment have synergistic effects on intestinal calcium absorption in rats fed a normal calcium diet (37). This is supported by the fact that the MPG-encoding gene incorporates the binding sites of vitamin D in its promoter regions (38). In humans, a meta-analysis of 8 RCTs has suggested that vitamin K combined with D significantly decreased ucOC and increased BMD (39). Further, a lack of vitamins D and K is associated with arterial hypertension (40), increased all-cause mortality risk and possibly cardiovascular mortality and cardiovascular events (41). Their supplementation is associated with a positive effect on carotid artery wall characteristics (42) and coronary artery calcium progression (43). However, this evidence is currently not robust and consistent enough to support co-administration of vitamin D and K in patients with osteoporosis or cardiovascular disease requiring further research (40).

Previous analyses in the single-center, double-blind, placebo-controlled, parallel-group Styrian Vitamin D Hypertension Trial in 200 participants, found no significant effect of vitamin D supplementation with 2,800 international units (IU) daily for 8 weeks on cardiovascular parameters (44) and bone turnover markers (45). However, the published evidence on vitamin K raises the question whether baseline vitamin K status—reflecting an individual’s dietary intake—would influence these findings. The hypothesis we aimed to evaluate was that beneficial effects of vitamin D supplementation are restricted to or pronounced in individuals with adequate vitamin K versus a poor vitamin K status.

The aim of this study was therefore to assess the effect of vitamin D supplementation on bone turnover and cardiovascular parameters depending on the vitamin K status at baseline (i.e., levels of vitamin K1, MK4 and MK7).

## Materials and methods

### Study design

This is a post-hoc analysis of the single-center, double-blind, placebo-controlled, parallel-group Styrian Vitamin D Hypertension Trial (44) carried out at the Medical University of Graz, Austria. The Styrian Vitamin D Hypertension Trial was registered at EU Clinical Trials Register (http://www.clinicaltrialsregister.eu, accessed 16 February 2011, EudraCT number 2009–018125–70) and at clinicaltrials.gov (ClinicalTrials.gov Identifier NCT02136771) and its publications adhere to the Consolidated Standards of Reporting Trials (CONSORT) 2010 statement (46).

### Study participants

Participants were adults aged 18 years or older with arterial hypertension (defined according to guidelines valid at the time of inclusion (47)) and a 25(OH)D serum concentration < 30 ng/mL (multiply by 2.496 to convert ng/mL to nmol/L). The study was performed between June 2011 and August 2014.

Exclusion criteria have been previously published (44) and include elevated levels of serum calcium, acute coronary syndrome or cerebrovascular events within the previous two weeks, pregnancy and lactation, drug intake due to participation in another clinical study or an estimated glomerular filtration rate <15 mL/min per 1.73 m^2^, a change in antihypertensive treatment during the previous four weeks or planned change of antihypertensive treatment, an estimated life expectancy of less than 12 months, 24-h systolic blood pressure (BP) > 160 mmHg or <120 mmHg, 24-h diastolic BP > 100 mmHg, any relevant acute diseases requiring drug therapy, chemotherapy or radiation, or a regular daily intake of more than 880 IU of vitamin D during the last four weeks in addition to the study medication. All participants gave their written informed consent prior to study inclusion. The ethics committee of the Medical University of Graz, Austria, approved the study, which was in line with the Declaration of Helsinki. Between June 2011 and August 2014, participants were recruited from the outpatient clinics of the Division of Cardiology and the Division of Endocrinology and Diabetology, Department of Internal Medicine, Medical University of Graz, Graz, Austria.

### Intervention

A web-based software (http://www.randomizer.at/, provided by the Institute for Medical Informatics, Statistics and Documentation, Medical University of Graz, Austria) was used to randomly allocate eligible participants in a 1:1 ratio to receive either 2,800 IU of vitamin D3 (Oleovit D3, Fresenius Kabi Austria, Austria), or a matching placebo (coconut oil). Both were administered orally by seven oily drops per day for 8 weeks. Patients returned the empty bottles at the follow-up study visits to ensure adequate intake. We performed a permuted block randomization with a block size of 10 and stratification according to gender. All investigators/authors enrolling participants, collecting data, and assigning intervention as well as the study participants were blinded to participant allocation.

Compliance in percent was measured according to the amount of study medication taken by the participant as a portion of the amount of the full study medication (12.5 mL) that was handed out at the baseline visit. The amount of study medication taken by the participant was calculated as 12.5 mL minus mL left-over at the end of the trial.

### Outcome measures

The primary outcome measures in this post-hoc analysis were the between-group differences in the bone turnover markers OC, bone specific alkaline phosphatase (bALP), C-terminal peptide (CTX), procollagen type 1 N-propeptide (P1NP), as well as the cardiovascular markers triglycerides, HbA1c, fasting glucose, HDL, LDL, total cholesterol, 24-h systolic and diastolic blood pressure, and CRP at study end with adjustment for the respective baseline levels.

Secondary, supplementary outcomes included NTproBNP, QTcBaz, renin, aldosterone, urinary albumin-to-creatinine ratio, HOMA-IR, pulse wave velocity, parathyroid hormone (PTH), alkaline phosphatase, calcium, phosphate, eGFR (CKD-EPI), vitamin D (25-OH), vitamin D (1,25-OH /calcitriol).

Vitamin K concentrations falling at or below compound-specific limits of detection (LOD; K1 = 0.02 nmol/L, MK-4 = 0.06 nmol/L, MK-7 = 0.04 nmol/L) were treated as left-censored. True missing values (no measurement attempt) were excluded from all downstream analyses. Moderators and outcomes with right-skewed distributions (all vitamin K measures, NTproBNP, CRP, renin, urinary albumin-to-creatinine ratio, triglycerides, pulse wave velocity, PTH, alkaline phosphatase, osteocalcin, glucose, HbA1c, aldosterone, and HOMA-IR) were log-transformed.

### Measurements

We performed all study visits with patient interviews, physical examinations, and sampling of blood between 7 a.m. and 11 a.m. after an overnight fast throughout the year. Ambulatory BP monitoring (ABPM) measurements (Spacelabs 90217A; Spacelabs Healthcare, Inc., Issaquah, WA) started after the visit following the recommendations of the European Society of Hypertension (48) and with good performance (49, 50). The next day, eligible patients were randomized and started taking the study medication. ABPM measurements were repeated after 8 weeks.

Serum levels of 25(OH)D were measured by a chemiluminescence assay (IDS-iSYS 25-hydroxyvitamin assay; Immunodiagnostic Systems Ltd., Boldon, UK) with an intra-assay and inter-assay coefficient of variation (CV) of 6.2 and 11.6%, respectively; plasma aldosterone concentration (PAC) by means of a RIA (Active Aldosterone RIA DSL-8600; Diagnostic Systems Laboratories, Inc., Webster, TX) with an intra-assay and inter-assay CV of 3.3 to 4.5% and 5.9 to 9.8%, respectively; plasma renin concentrations (PRC) by a “RENIN III GENRATION” (GEN. III) RIA assay (Renin IRMA RIA-4541, DRG Instruments GmbH, Marburg, Germany) with an intra-assay and inter-assay CV of 0.6 to 4.5% and 2.7 to 14.5%, respectively. All other parameters were determined by routine laboratory procedures. All parameters were measured daily.

Total OC (intra and interassay CV, 0.5 and 1.4%, respectively; analytical range, 0.5–300 ng/mL) was measured by electrochemiluminescence immunoassay (Roche Diagnostics, Mannheim, Germany) according to the manufacturer’s instructions detecting all forms of OC with a similar affinity; CTX (intra and inter-assay CV, 2.0 and 4.2%, respectively; analytical range, 0.01–6 ng/mL) by electrochemiluminescence immunoassay (Cobas, Roche Diagnostics, Mannheim, Germany); bALP (interassay CV: 5.2%) by means of a spectrophotometric immunoassay (IDS-ISYS Ostase BAP; Immunodiagnostic Systems Ltd. [IDS Ltd.], Boldon, Tyne & Wear, UK); P1NP by (interassay CV: 2.7%) means of an automated electrochemiluminescence immunoassay (ECLIA; Roche Diagnostics, Mannheim, Germany); 1,25(OH)2D3 by chemiluminescence immunoassay (IDS-iSYS 1,25VitDXp; Immunodiagnostic Systems Ltd., Boldon, UK) with an intra-assay and inter-assay CV of 6.4–12.1% and 6.6–9.6%, respectively.

In serum samples that were stored at −80° Celsius from blood collection until analysis, vitamin K1, MK-4 and MK-7 were measured with a liquidchromatography tandem mass spectrometric (LC–MS/MS) method developed by Meinitzer et al. (51, 52). After protein precipitation, extraction and separation, the samples were ionized by atmospheric pressure ionisation (APCI) and analysed with the SCIEX QTRAP 6500 liquid chromatography mass spectrometer (LCMS) (Applied Biosystems, Framingham, MA, United States). Limit of detection and limit of quantification of all vitamin K analytes ranged from 0.004 to 0.006 nmol/L and from 0.012 to 0.015 nmol/L. Imprecisions of all analytes (intra and interday CVs) ranged between 4.8 and 17.7%.

### Statistical methods

Sample size calculations were based on the primary outcome of the trial, as published previously (44). Continuous data following a normal distribution are shown as means with standard deviations, variables with a non-normal distribution are shown as medians with interquartile ranges (IQR) and categorical data as percentages. Group comparisons at baseline were analyzed with either unpaired Student’s *t*-tests or chi-squared tests or, in case of a non-normal distribution, by use of the Mann–Whitney U test.

Data included multiple measures associated with bone and cardiovascular health at baseline and follow-up (outcome measures). Blood samples were subsequently analyzed to assess three vitamin K related moderators: Vitamin K1, MK-4 and MK-7.

The relationships between vitamin K1, MK-4, and MK-7 at baseline were explored via Spearmen correlations, as were the relationships with vitamin K and bone as well as cardiovascular parameters. For this, data of all screened individuals were used, independent of whether they were eligible for randomization (44). We also investigated whether supplementation had an effect on parameters of vitamin K using ANCOVA (adjusted for respective baseline levels, age, sex, and BMI).

The main analytical approach for moderation analyses differed by marker, reflecting the extent of left-censoring in each. For vitamin K1 and MK-7, which had moderate censoring rates, a quantile regression-based imputation under a left-censored normal distribution (QRILC; (53)) was applied to the log-transformed moderator values. This procedure was repeated across *m* = 100 imputed datasets, and results were pooled using Rubin’s rules. For MK-4, which exhibited more than 70% censoring, the moderator was dichotomized into detectable versus undetectable categories and used as a binary variable in a single-dataset complete-case analysis; no imputation was performed.

All moderation analyses used ANCOVA models and corrected for baseline outcome, age, sex and BMI, with Vitamin K value as a moderator (interaction) of the treatment group (vitamin D or placebo). The interaction term was the term of interest. Heteroscedasticity-consistent standard errors (HC3) were applied throughout using the sandwich package. For QRILC analyses, interaction estimates from each imputed dataset were pooled using Rubin’s rule (54).

For each marker, two supplementary analyses were run on the primary outcomes to assess the robustness of the main findings. 1) All markers were rerun for detectable-only moderator values. 2) For K1 and MK-7 (main: QRILC), supplementary analyses using a binary detectable/undetectable moderator were run, while MK-4 (main: binary) was analysed using the described QRILC approach. Additionally, the respective main analyses were also run on the supplementary outcomes.

To account for multiple testing, for the primary outcomes, Holm’s method and the Benjamini-Hochberg false discovery rate (FDR) procedure were applied separately within each marker (12 tests).

All analyses were conducted in R (version: 4.4.2) (R Core Team: A Language and Environment for Statistical Computing. R Foundation for Statistical Computing; Available from: https://www.R-project.org/).

## Results

**Figure 1:**
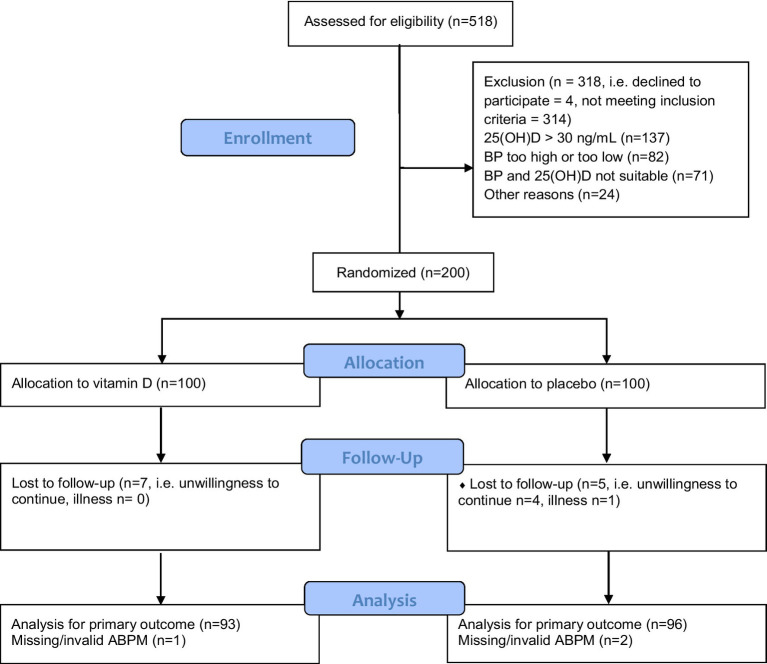
Study design and participant flowchart.

Two-hundred patients were randomized (see Figure 1), 188 completed the study with a mean age of 60.1 (standard deviation 11.3) years; 47% were women. In the vitamin D group, C-reactive protein was slightly higher than in the placebo group, while N-terminal pro-B-type natriuretic peptide was slightly higher in the placebo group. Apart from these two parameters, no other characteristics differed between the vitamin D and placebo group. In our data, the leftover in the bottles was below or equal to 15% of the 12,5 mL of the bottle content in 87% of participants. Baseline characteristics of patients are shown in Table 1.

**Table 1:** Baseline characteristics of study participants eligible for randomization.

Baseline characteristics	All (*n* = 197)	Vitamin D (*n* = 98)	Placebo (*n* = 99)	*p*-value
Age (years)	60.1 ± 11.0	60.4 ± 10.9	59.7 ± 11.2	0.660
Females (%)	47	47	47	0.827
BMI (kg/m^2^)	29.7 (27.2–32.8)	29.6 (27.5–32.9)	29.8 (26.5–32.8)	0.622
24 h systolic BP (mmHg)	131.7 ± 9.3	131.8 ± 8.8	131.7 ± 9.7	0.943
24 h diastolic BP (mmHg)	78.1 ± 7.9	78.5 ± 7.6	77.8 ± 8.3	0.547
Vitamin K1 (nmol/L)	0.9 (0.6–1.5)	0.9 (0.5–1.4)	1.0 (0.6–1.7)	0.379
MK4 (nmol/L)	0.1 (0.1–0.2)	0.1 (0.1–0.2)	0.1 (0.1–0.2)	0.970
MK7 (nmol/L)	0.8 (0.5–1.1)	0.7 (0.5–1.1)	0.8 (0.5–1.0)	0.865
PTH (pg/mL)	49.1 (39.5–63.4)	48.7 (39.5–60.0)	51.5 (39.6–64.9)	0.666
BAP (μg/L)	15.9 (13.1–20.1)	15.3 (12.3–18.9)	16.6 (13.6–20.7)	0.097
CTX (ng/mL)	0.2 ± 0.2	0.2 ± 0.2	0.2 ± 0.2	0.605
Osteocalcin (ng/mL)	12.4 (9.1–16.8)	11.9 (9.0–16.6)	13.0 (9.4–17.9)	0.235
P1NP (ng/mL)	40.9 ± 18.8	38.7 ± 17.5	43.0 ± 19.8	0.114
Serum calcium (mmol/L)	2.4 ± 0.1	2.4 ± 0.1	2.4 ± 0.1	0.683
Serum phosphate (mg/dL)	2.9 ± 0.5	2.9 ± 0.5	3.0 ± 0.5	0.108
NT-proBNP (pg/mL)	82.0 (41.0–148.0)	65.0 (35.2–136.8)	98.0 (55.0–162.0)	0.049
QTc Bazett (ms)	418.4 ± 35.8	418.4 ± 37.8	418.5 ± 34.0	0.983
Pulse wave velocity (m/s)	8.3 (7.1–9.3)	8.2 (7.4–9.2)	8.3 (7.0–9.6)	0.941
Renin (μU/mL)	15.3 (8.2–36.7)	15.6 (8.6–36.0)	14.8 (7.4–37.2)	0.718
Aldosterone (ng/dL)	7.2 (4.4–10.8)	6.2 (3.9–10.4)	8.6 (5.4–11.6)	0.056
Total cholesterol (mg/dL)	197.2 ± 52.0	197.9 ± 46.7	196.6 ± 56.9	0.852
Fasting glucose (mg/dL)	100.0 (92.0–131.0)	101.0 (94.2–127.2)	99.0 (89.0–133.0)	0.559
HbA1c (%)	5.8 (5.5–6.8)	5.8 (5.4–6.6)	5.8 (5.5–6.9)	0.350
HOMA-IR	2.0 (1.2–3.7)	2.0 (1.2–3.6)	1.8 (1.1–3.7)	0.748
25(OH)D (ng/mL)	21.1 ± 5.6	21.8 ± 5.5	20.3 ± 5.7	0.057
1,25(OH)2D3 (pg/mL)	49.8 ± 18.8	51.0 ± 18.5	48.5 ± 19.2	0.362
HDL-cholesterol (mg/dL)	56.5 ± 16.7	56.0 ± 16.9	56.9 ± 16.5	0.706
LDL-cholesterol (mg/dL)	114.0 ± 39.9	115.8 ± 40.8	112.2 ± 39.0	0.527
Triglycerides (mg/dL)	119.0 (78.5–163.5)	119.0 (73.5–160.0)	117.5 (83.8–168.5)	0.610
eGFR CKD-EPI (mL/min/1.73 m^2^)	82.3 ± 17.8	83.4 ± 17.1	81.2 ± 18.4	0.387
Urinary albumin/creatinine (mg/g)	11.0 (5.0–24.0)	9.5 (4.2–20.0)	12.0 (5.0–33.5)	0.214
CRP (mg/L)	1.9 (0.9–3.6)	2.4 (1.2–3.8)	1.4 (0.9–3.3)	0.035

Baseline characteristics of Vitamin K1, MK-4 and MK-7 were only based on values above the lower level of detection (LOD). This includes 193, 59 and 176 individuals in Vitamin K1, MK-4, and MK-7, respectively. Data are shown as means with standard deviation, median, and interquartile ranges, or as percentages, as appropriate. Comparisons between vitamin D and placebo groups were performed by a Student’s t-test, a Mann–Whitney U-test, or a chi-square test. BMI, body-mass index; BP, blood pressure; MK-4, menaquinone 4; MK-7, menaquinone 7; PTH, parathyroid hormone; bALP, bone-specific alkaline phosphatase; CTX, β-CrossLaps; P1NP, procollagen type 1 amino-terminal propeptide; NT-proBNP, N-terminal pro-B-type natriuretic peptide; HbA1c, glycated hemoglobin; HOMA-IR, homeostasis model assessment-insulin resistance; 25(OH)D, 25-hydroxyvitamin D; 1,25(OH)2D3, calcitriol; HDL-cholesterol, high-density lipoprotein-cholesterol; LDL-cholesterol, low-density lipoprotein-cholesterol; eGFR, estimated glomerular filtration rate; CRP, C-reactive protein.

There was no excess of adverse events (ie, hypercalcemia or hospitalizations) in the vitamin D group, as published in the main manuscript of the trial (44). Of the 200 individuals randomized in this trial, 19 had self-reported osteoporosis, 6 were on bisphosphonates, one on denosumab. In the previously published main trial, there was no significant effect of vitamin D supplementation on the primary outcome parameter 24-h systolic BP with a mean treatment effect (95% confidence interval [CI]) of −0.4 (−2.8 to 1.9) mm Hg (*p* = 0.712) (44). We observed a significant increase in 25(OH)D and a significant decrease in PTH, which has been published previously (44). With regards to mineral metabolism, 25(OH)D significantly increased over the study period with a mean treatment effect [95% CI] of 11.5 [9.4–13.7] ng/mL, *p* < 0.001, and PTH significantly decreased (with a treatment effect of −5.7 [−9.3 to −2.1] pg./mL, *p* = 0.003) (44).

Moderation analyses showed no significant interaction between vitamin K status and intervention condition (vitamin D supplementation/placebo) on parameters of bone metabolism and cardiovascular parameters after correcting for multiple comparisons (see Table 2), i.e., no interaction remained significant after correction. This was applicable for both Bonferroni-Holm and FDR corrections. In order to perform moderation analyses, a quantile regression-based imputation under a left-censored normal distribution (QRILC; (53))—a preferred method for imputing left-censored, i.e., not detected, values – was applied to the log-transformed moderator values K1 and MK-7, as these parameters had moderate censoring rates. Given the expected high proportion of values below the lower limit of detection, MK-4 was dichotomized into detectable versus undetectable categories and used as a binary variable; no imputation was performed. In sensitivity analysis, when performing QRILC imputation for MK-4 and dichotomization for vitamin K1 and MK-7, results remained materially unchanged (data not shown).

**Table 2:** Main moderator analyses of K1 (QRILC), MK-7 (QRILC) and MK-4 (Binary) moderators and bone turnover markers and cardiovascular outcome measures.

Outcome	K1 (QRILC)							
	*n*	Vitamin D/placebo	*β*	SE	FMI	p (raw)	p (holm	p (fdr)
Triglyzeride	159	77/82	0.16	0.07	0.005	0.016	0.188	0.188
24 h systolic BP	179	89/90	3.33	1.54	0.005	0.031	0.344	0.188
HDL	183	90/93	−1.67	4.01	<0.001	0.254	1.000	0.609
LDL	175	85/90	5.08	1.12	0.008	0.206	1.000	0.609
24 h diastolic BP	179	89/90	1.36	1.47	0.002	0.226	1.000	0.609
CRP	182	90/92	−0.12	3.13	0.002	0.410	1.000	0.703
P1NP	160	77/83	−2.82	0.15	0.003	0.368	1.000	0.703
CTX	148	70/78	0.01	6.85	0.021	0.641	1.000	0.855
Total cholesterol	183	90/93	3.59	0.03	0.002	0.600	1.000	0.855
HbA1c	110	52/58	−0.00	0.07	0.007	0.884	1.000	0.884
Glucose	183	90/93	0.01	0.03	0.003	0.861	1.000	0.884
OC	167	81/86	0.02	0.01	0.002	0.760	1.000	0.884

Outcome	MK-7 (QRILC)							
	n	Vitamin D/placebo	*β*	SE	FMI	*p* (raw)	*p* (holm	*p* (fdr)
24 h systolic BP	179	89/90	3.27	1.32	0.016	0.013	0.162	0.162
OC	167	81/86	−0.12	0.05	0.021	0.027	0.301	0.164
HbA1c	110	52/58	0.02	0.04	0.005	0.291	1.000	0.569
HDL	183	90/93	1.25	2.81	0.014	0.388	1.000	0.569
LDL	175	85/90	2.75	4.62	0.016	0.427	1.000	0.569
Glucose	183	90/93	0.05	0.02	0.076	0.201	1.000	0.569
P1NP	160	77/83	−3.53	0.03	0.019	0.210	1.000	0.569
CTX	148	70/78	−0.03	1.45	0.014	0.372	1.000	0.569
Total cholesterol	183	90/93	5.20	3.46	0.015	0.261	1.000	0.569
Triglyceride	159	77/82	−0.03	0.06	0.009	0.556	1.000	0.668
24 h diastolic BP	179	89/90	0.37	0.79	0.011	0.643	1.000	0.701
CRP	182	90/92	0.03	0.12	0.023	0.826	1.000	0.826

Outcome	MK-4 (Binary)							
	*n*	Vitamin D/placebo	Det. *n*	*β*	SE	*p* (raw)	p (holm	*p* (fdr)
24 h systolic BP	182	90/92	52 det. / 130 non-det.	−4.83	1.72	0.062	0.683	0.373
24 h diastolic BP	182	90/92	52 det. / 130 non-det.	−3.43	2.57	0.048	0.576	0.373
LDL	178	86/92	53 det. / 125 non-det.	−8.72	5.66	0.125	1.000	0.388
Glucose	186	91/95	54 det. / 132 non-det.	−0.09	0.06	0.129	1.000	0.388
HDL	186	91/95	54 det. / 132 non-det.	4.49	3.25	0.170	1.000	0.407
Triglyceride	161	78/83	51 det. / 110 non-det.	−0.10	0.12	0.385	1.000	0.608
HbA1c	113	53/60	54 det. / 131 non-det.	−0.02	0.24	0.456	1.000	0.608
CRP	185	91/94	51 det. / 119 non-det.	−0.21	0.11	0.392	1.000	0.608
OC	170	82/88	32 det. / 81 non-det.	0.09	0.03	0.414	1.000	0.608
P1NP	162	77/85	51 det. / 111 non-det.	−2.45	5.27	0.643	1.000	0.771
CTX	150	70/80	44 det. / 106 non-det.	−0.02	0.05	0.772	1.000	0.842
Total cholesterol	186	91/95	54 det. / 132 non-det.	1.20	10.54	0.910	1.000	0.910

All moderation analyses were corrected for baseline values, age, sex and BMI. P-value corrections are performed for each marker indepentently. Interaction estimates for continuous vitamin K moderators were derived from m = 100 QRILC-imputed datasets and pooled using Rubin’s rules. *β*, pooled interaction coefficient; SE, standard error; FMI, fraction of missing information, the proportion of uncertainty in the pooled estimate attributable to QRILC imputation., Det., detectable. *p*-values were adjusted for multiple comparisons using both Bonferroni-Holm (more conservative) and false discovery rate (FDR; less conservative) corrections. MK-4, menaquinone 4; MK-7, menaquinone 7; BMI, body mass index; BP, blood pressure; LDL-cholesterol, low-density lipoprotein-cholesterol; HDL-cholesterol, high-density lipoprotein-cholesterol; CTX, β-CrossLaps; P1NP, procollagen type 1 amino-terminal propeptide; OC, osteocalcin; HbA1c, glycated haemoglobin; CRP, C-reactive protein.

**Figure 2:**
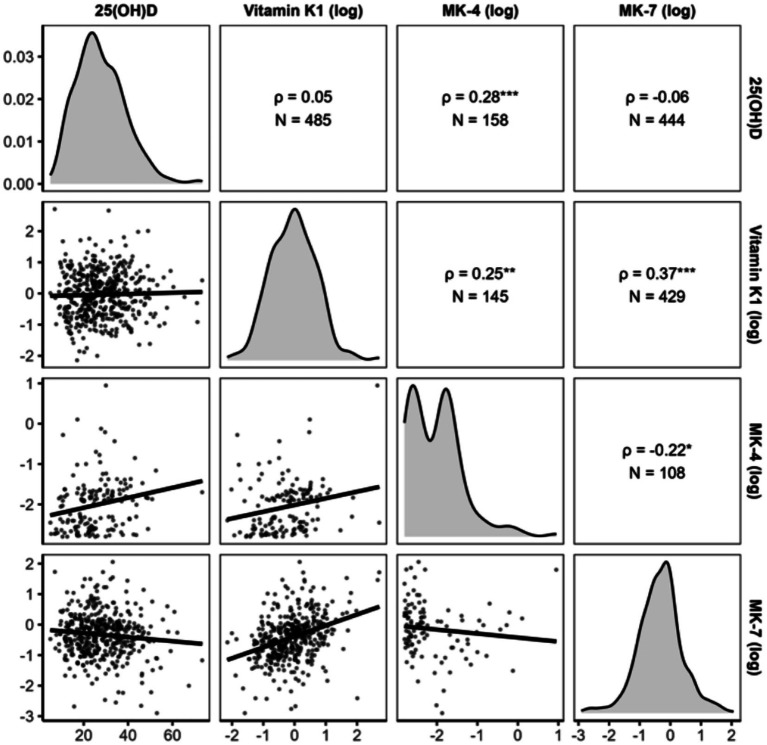
Relationships between vitamin K1, MK-4 and MK-7 at baseline. Relationships between vitamin K1, MK-4 and MK-7 at baseline were explored via Spearmen correlations. Data of all screened individuals were used, independent of whether they were eligible for randomization. Vitamin K1, MK4, and MK7 correlated significantly with each other. 25(OH)D correlated only with MK-4. 25(OH)D, 25-hydroxyvitamin D; MK-4, menaquinone 4; MK-7, menaquinone 7; epoxide, vitamin K1 2,3-epoxide; *** = *p* < 0.001.

Vitamin K1, MK4, and MK7 correlated significantly with each other. 25(OH)D correlated only with MK-4 (Figure 2). In exploratory analyses, vitamin K1 correlated with CTX and triglycerides. MK-4 correlated only with HbA1c, while MK-7 correlated weakly with fasting glucose, HOMA IR, and triglycerides. However, the observed correlations were weak and could not be observed consistently across all parameters of vitamin K and—as for glucose and HOMA IR—across all parameters of glucose metabolism (see Supplementary Table S1, FDR correction shown, Bonferroni not shown).

ANCOVA showed no treatment effect of vitamin D on any vitamin K measurement (Table 3).

**Table 3:** Vitamin K measurements at baseline and at follow-up, as well as ANCOVA results adjusted for baseline, sex, age and BMI.

	Vitamin D effects on vitamin K1, MK-4 and MK-7			
	Baseline	Follow-up	Treatment effect (log scale)	*p*-Value
Vitamin K1 (ng/mL)				
Treatment (*n* = 83)	0.932 (0.571–1.485)	1.000 (0.669–1.595)	0.147 (−0.031 to 0.324)	0.105
Placebo (*n* = 85)	0.926 (0.598–1.620)	0.912 (0.591–1.370)		
MK-4 (pg/mL)				
Treatment (*n* = 12)	0.178 (0.068–0.197)	0.162 (0.093–0.372)	0.270 (−0.430 to 0.970)	0.430
Placebo (*n* = 14)	0.155 (0.091–0.190)	0.118 (0.078–0.266)		
MK-7 (pg/mL)				
Treatment (*n* = 72)	0.730 (0.479–1.100)	0.634 (0.468–0.883)	0.113 (−0.105 to 0.331)	0.307
Placebo (*n* = 83)	0.767 (0.465–1.040)	0.576 (0.386–0.804)		

Data are shown as median with interquartile ranges. Treatment effects with 95% confidence interval and p-values were calculated by ANCOVA with adjustment for baseline values, sex, age and BMI. MK-4, menaquinone 4; MK-7, menaquinone 7; BMI, body mass index.

## Discussion

In this post-hoc analysis from the Styrian Vitamin D Hypertension Trial, we observed no vitamin K 1-, MK4 or MK7-status dependent effect of vitamin D supplementation on the bone turnover markers OC, CTX, and P1NP or on cardiovascular parameters. We did not find a significant effect of vitamin D supplementation on vitamin K 1, MK4 or MK7. In further exploratory analyses, some parameters of vitamin K status correlated weakly and inconsistently with certain bone or cardiovascular parameters.

In our post-hoc analyses from a short randomized controlled trial using LC/MS measurement of vitamin K, we were thus not able to show what previous data from genetic analyses (38) or a prospective observational study on mortality have previously suggested: a synergistic effect of the vitamins D and K, i.e., that a person’s vitamin K status, mainly influenced by diet, would alter the response to vitamin D supplementation. These findings might either be due to a true lack of an effect or mirror current limitations of vitamin D interventional research. Despite strong mechanistic and observational data, large RCTs could not confirm clear cardiovascular benefits of vitamin D. So similarly, possible explanations for a lack of synergistic vitamin D and K effects could be methodological aspects, treatment duration, etc. (55).

Our LC/MS analyses yielded plausible values of vitamin K1, MK-4 and MK-7 as they are in accordance with previously suggested reference ranges for European cohorts (56). The high proportion of undetectable MK-7 levels in this European cohort is expected due to the limited MK-7 content in our diet as opposed to Japanese individuals (57). Significant amounts of MK-4 are only detectable in relevant amounts in persons with MK-4 supplementation (57), i.e., in around 26% of individuals, as MK-4 is predominantly synthesized endogenously from dietary vitamin K1 (57). Our data are well in line with these findings, affirming the plausibility of our results.

In support of our moderation results, vitamin K in combination with vitamin D and calcium had no significant effect on the bone turnover markers P1NP, CTX, bALP and OC as compared to placebo in postmenopausal women with osteopenia over 3 years (58). Likewise, CTX and P1NP did not differ significantly following either K1 or MK-4 supplementation compared to placebo in 105 women over 18 months (59). In contrast, several studies analyzing vitamin K supplementation effects alone or in combination with vitamin D or other supplements on BMD have shown moderate effects in reducing bone loss (60–62), increasing BMD (39) or a preservation of trabecular bone structure at the tibia for MK-7 versus placebo (63). Unfortunately, BMD and HRpQCT data for the present cohort were not available.

As with bone turnover markers, we did not observe a significant interaction of vitamin K status with vitamin D supplementation on cardiovascular markers. Studies showing potential for the combined effect of vitamins D + K versus D alone on cardiovascular risk markers are limited by the lack of vitamin K measurement or the use of dp-ucMGP as a proxy for vitamin K deficiency (42, 43), as opposed to the use of LC/MS-derived data applied in our study. Other studies have shown effects of lower coronary artery calcium progression in very adherent patients only suggesting a weak effect of vitamins D and K (42, 43). A prospective cohort study indicated that low vitamin D in combination with high dp-ucMGP as a proxy for low vitamin K status was associated with increased systolic and diastolic blood pressure and a trend for higher incident arterial hypertension after 6 years of follow-up (40). Interactions for incident arterial hypertension, though, were non-significant, potentially owing to the small sample size – this lacking association with blood pressure is in line with our findings. Comparing results across studies warrants caution due to the use of data-driven cutoff points for vitamin K status resulting from a lack of a clinically accepted dp-ucMGP cutoff points for healthy populations. More research is therefore needed to define optimal concentrations for vitamin K status using studies with long-term follow-up of clinical outcomes (40).

Our findings showing no effect of vitamin D on vitamin K are supported by a 2-year data in elderly women (64) and 12-month data in healthy Danish girls, showing that vitamin D supplementation does not affect serum %ucOC (65). In contrast, a 1-year supplementation of calcium and vitamin D did cause a decrease in ucOC in 195 elderly institutionalized women (66). Of note, our study is, to our knowledge, the first to analyze the effect of vitamin D on vitamin K status as measured by LC–MS in contrast to measuring its proxy ucOC.

In our analysis, we found only weak and inconsistent correlations of vitamin K status parameters with certain bone and cardiovascular parameters that have to be cautiously interpreted due to multiple testing (though corrections were performed) and should only be regarded as hypothesis generating.

Strengths of our study include the evaluation of possible interactions of vitamin K and vitamin D as well as the LC/MS method, which is a validated form of measuring vitamin K. Determining vitamin K status is a more objective measure of nutrient status compared to dietary intake alone or using the proxy parameter ucOC. The method of measurement by means of LCMS has been well validated, previously published (51, 52) and is the most reliable available to date. It is considered the gold standard for determining small molecular analytes.

The importance of an adequate LCMS measurement of vitamin K is highlighted by its lipophilic nature and very low concentrations (51) as well as the short half-life of vitamin K1 of only 1–3 h and its dependence on the intake of leafy vegetables in the days prior to measurement hours (67). Menaquinones are also highly dependent on the intake of certain foods (67). They are mostly produced by intestinal gut microbiota and are located in bacterial membranes to a large extent. Data on the amount of menaquinones that are actually absorbed from the gut are currently still sparse (67). As menaquinone content in food differs according to the geographical location studied, MK-4 is the only one detectable in Western diet. Without natto intake, MK-7 is often below the limit of detection due to very low concentrations (68), as reflected by our data.

One limitation is that we did not assess participants’ dietary vitamin K intake by means of questionnaires, to understand the effects of dietary habits on LC/MS-measured vitamin K parameters. This would add valuable information to the field of vitamin K research in future trials. Further limitations of our trial include the fact that we did not assess fracture risk in our patients. Previous fractures could have an impact on bone turnover markers and possible influence its susceptibility to vitamin K status. Further, it is currently unknown how to define vitamin K deficiency due to the lack of a common consensus on vitamin K status classification. In addition, currently used assay methodologies differ substantially. Our analyses may also be partially restricted by its sample size and multiple testing. We tried to address this issue by implementing FDR, a very soft correction for multiple testing. Importantly, even in the uncorrected outcomes, no values had a *p*-value below 0.02, which suggests that even extremely liberal corrections or a reduction in tests would not have led to significant interaction terms. Further, this post-hoc analysis was exploratory and hypothesis-generating, aiming to assess effect modification rather than to test a pre-specified primary hypothesis. Also, as the intervention period was relatively short, the absence of intervention effects could partly reflect insufficient duration rather than a true lack or biological interaction. Lastly, given its short duration, our study has focused on biochemical and surrogate endpoints rather than hard clinical endpoints such as fractures or cardiovascular events. As such, translatability of our results into clinical practice may be limited.

Taken together, published data provide heterogeneous evidence of an association of vitamin K status and vitamins K and D co-treatment on surrogate markers of bone and cardiovascular health. A consistent evidence of a beneficial effect of vitamin K status alone or the interaction between vitamin D and K is lacking and vitamin K intake is therefore not supported by current guidelines. Our data, derived from a post-hoc analysis and exploratory by nature, underscore this approach and do not provide evidence that a sufficient vitamin K status or supply modifies or enhances effects of vitamin D supplementation. Our results do not provide evidence in support of the co-administration of vitamin D with vitamin K to improve bone or cardiovascular health.

Conclusive evidence on the relationship between vitamins D and K will require further trials, both on surrogate markers of bone and cardiovascular health, as well as large osteologic and cardiovascular outcome trials over longer follow-up periods to evaluate the potential benefits of vitamin K supplementation and its combination with vitamin D.

## Data Availability

The raw data supporting the conclusions of this article will be made available by the authors, without undue reservation.
