# Supplementary material for: Vitamin K status has no influence on the effect of vitamin D supplementation on bone turnover and cardiovascular markers: a randomized controlled trial
Source: Front Nutr. 2026 Jun 30;13:1857443. doi: 10.3389/fnut.2026.1857443 (PMC13365057; doi:10.3389/fnut.2026.1857443)
Supplement: Supplementary file 1 [file Table_1.docx]

| **Laboratory parameters** | **Vitamin K1** | **MK-4** | **MK-7** |
| --- | --- | --- | --- |
| AP (µg/L) | ρ = -0.02 (p_FDR = 0.698), N = 484 | ρ = -0.05 (p_FDR = 0.672), N = 158 | ρ = 0.05 (p_FDR = 0.515), N = 443 |
| Aldosterone (ng/dL) | ρ = 0.04 (p_FDR = 0.651), N = 314 | ρ = 0.03 (p_FDR = 0.735), N = 118 | ρ = 0.15 (p_FDR = 0.043), N = 284 |
| BMI (kg/m²)I | ρ = 0.05 (p_FDR = 0.515), N = 478 | ρ = 0.06 (p_FDR = 0.642), N = 156 | ρ = 0.10 (p_FDR = 0.104), N = 438 |
| CRP (mg/L) | ρ = 0.02 (p_FDR = 0.710), N = 484 | ρ = -0.05 (p_FDR = 0.672), N = 158 | ρ = 0.10 (p_FDR = 0.109), N = 443 |
| CTX (ng/mL) | ρ = -0.17 (p_FDR = 0.006), N = 430 | ρ = 0.07 (p_FDR = 0.627), N = 139 | ρ = -0.12 (p_FDR = 0.068), N = 398 |
| 1,25(OH)2D3 (pg/mL) | ρ = 0.02 (p_FDR = 0.698), N = 478 | ρ = 0.08 (p_FDR = 0.515), N = 157 | ρ = 0.03 (p_FDR = 0.642), N = 438 |
| Serum calcium (mmol/L) | ρ = 0.05 (p_FDR = 0.515), N = 485 | ρ = 0.10 (p_FDR = 0.457), N = 158 | ρ = 0.05 (p_FDR = 0.515), N = 444 |
| Total cholesterol (mg/dL) | ρ = 0.12 (p_FDR = 0.047), N = 485 | ρ = 0.17 (p_FDR = 0.100), N = 158 | ρ = 0.15 (p_FDR = 0.012), N = 444 |
| Fasting glucose (mg/dL) | ρ = 0.06 (p_FDR = 0.382), N = 485 | ρ = -0.15 (p_FDR = 0.179), N = 158 | ρ = 0.18 (p_FDR = 0.003), N = 444 |
| HDL-cholesterol (mg/dL) | ρ = -0.15 (p_FDR = 0.009), N = 484 | ρ = -0.00 (p_FDR = 0.988), N = 157 | ρ = -0.13 (p_FDR = 0.040), N = 443 |
| HOMA-IR | ρ = 0.12 (p_FDR = 0.043), N = 483 | ρ = 0.00 (p_FDR = 0.988), N = 158 | ρ = 0.20 (p_FDR = 0.001), N = 442 |
| HbA1c (%) | ρ = 0.08 (p_FDR = 0.259), N = 480 | ρ = -0.28 (p_FDR = 0.005), N = 157 | ρ = 0.13 (p_FDR = 0.040), N = 439 |
| LDL-cholesterol (mg/dL) | ρ = 0.05 (p_FDR = 0.489), N = 472 | ρ = 0.18 (p_FDR = 0.080), N = 156 | ρ = 0.08 (p_FDR = 0.295), N = 433 |
| NT-proBNP (pg/mL) | ρ = -0.07 (p_FDR = 0.275), N = 485 | ρ = -0.06 (p_FDR = 0.627), N = 158 | ρ = -0.03 (p_FDR = 0.642), N = 444 |
| Osteocalcin (ng/mL) | ρ = -0.12 (p_FDR = 0.040), N = 472 | ρ = 0.04 (p_FDR = 0.698), N = 154 | ρ = -0.06 (p_FDR = 0.489), N = 434 |
| P1NP (ng/mL) | ρ = -0.07 (p_FDR = 0.321), N = 463 | ρ = 0.17 (p_FDR = 0.115), N = 153 | ρ = -0.11 (p_FDR = 0.087), N = 427 |
| PTH (pg/mL) | ρ = 0.04 (p_FDR = 0.627), N = 485 | ρ = 0.05 (p_FDR = 0.667), N = 158 | ρ = -0.03 (p_FDR = 0.642), N = 444 |
| Pulse wave velocity (m/s) | ρ = 0.13 (p_FDR = 0.040), N = 422 | ρ = 0.05 (p_FDR = 0.667), N = 135 | ρ = 0.14 (p_FDR = 0.039), N = 388 |
| Serum phosphate (mg/dL) | ρ = 0.02 (p_FDR = 0.698), N = 485 | ρ = 0.06 (p_FDR = 0.642), N = 158 | ρ = 0.01 (p_FDR = 0.845), N = 444 |
| QTc Bazett (ms) | ρ = -0.14 (p_FDR = 0.020), N = 464 | ρ = -0.04 (p_FDR = 0.698), N = 148 | ρ = -0.05 (p_FDR = 0.515), N = 425 |
| Renin (µU/mL) | ρ = -0.12 (p_FDR = 0.109), N = 314 | ρ = -0.30 (p_FDR = 0.009), N = 118 | ρ = 0.04 (p_FDR = 0.656), N = 284 |
| 24h diastolic BP (mmHg) | ρ = 0.04 (p_FDR = 0.611), N = 484 | ρ = 0.19 (p_FDR = 0.065), N = 158 | ρ = -0.04 (p_FDR = 0.580), N = 443 |
| 24h systolic BP (mmHg) | ρ = 0.03 (p_FDR = 0.648), N = 485 | ρ = 0.09 (p_FDR = 0.489), N = 158 | ρ = -0.02 (p_FDR = 0.698), N = 444 |
| Triglycerides (mg/dL) | ρ = 0.35 (p_FDR = 0.000), N = 465 | ρ = 0.06 (p_FDR = 0.627), N = 152 | ρ = 0.36 (p_FDR = 0.000), N = 425 |
| Urinary albumin/creatinine (mg/g) | ρ = 0.06 (p_FDR = 0.489), N = 421 | ρ = 0.09 (p_FDR = 0.525), N = 139 | ρ = 0.04 (p_FDR = 0.627), N = 380 |
| 25(OH)D (ng/mL) | ρ = 0.05 (p_FDR = 0.504), N = 485 | ρ = 0.28 (p_FDR = 0.005), N = 158 | ρ = -0.06 (p_FDR = 0.489), N = 444 |
| eGFR CKD-EPI (mL/min/1.73 m²) | ρ = -0.04 (p_FDR = 0.627), N = 485 | ρ = -0.03 (p_FDR = 0.732), N = 158 | ρ = -0.06 (p_FDR = 0.415), N = 444 |

**Supplementary table 1: Correlations between vitamin K1, MK-4 and MK-7 and parameters of glucose and lipid metabolism**

BMI = body-mass index; BP = blood pressure; MK-4 = menaquinone 4; MK-7 = menaquinone 7; PTH = parathyroid hormone; bALP = bone-specific alkaline phosphatase; CTX = β-CrossLaps; P1NP = procollagen type 1 amino-terminal propeptide; NT-proBNP = N-terminal pro-B-type natriuretic peptide; HbA1c = glycated hemoglobin; HOMA-IR = homeostasis model assessment-insulin resistance; 25(OH)D = 25-hydroxyvitamin D; 1,25(OH)2D3 = calcitriol; HDL-cholesterol = high-density lipoprotein-cholesterol; LDL-cholesterol = low-density lipoprotein-cholesterol; eGFR = estimated glomerular filtration rate; CRP = C-reactive protein.
